# Supplementary material for: The General Population’s Inappropriate Behaviors and Misunderstanding of Antibiotic Use in China: A Systematic Review and Meta-Analysis
Source: Antibiotics (Basel). 2021 Apr 26;10(5):497. doi: 10.3390/antibiotics10050497 (PMC8146421; doi:10.3390/antibiotics10050497)
Supplement: Supplementary file 1 [file antibiotics-10-00497-s001.zip › antibiotics-1182592-supplementary.pdf]

Table S1. Search Strategy

Database	Search Strategy (Up to February)
CNKI (China National Knowledge Infrastructure)	SU = 'Antibacterial drugs' +'Antibiotics' +'Antimicrobial drugs ' AND TKA= 'Application' +'Management '+'Use' +'Storage' +'Purchase medicine' +'Purchase medicine without prescription' + 'Purchase medicine by yourself 'AND TKA='behavior' + 'status quo' + 'behavioral factors' + 'cognition' + 'attitude' + 'reasonable' + 'irrational' +'abuse' + 'expectation' +'medical advice' + 'adherence ' AND TKA = 'Resident' + 'Crowd' + 'General population' + 'Public' + 'Human' + 'Respiratory tract infection patients' +'Middle-aged and elderly' + 'students' + 'outpatients' (In Chinese)
WANFANG	(Chinese and English expansion & subject heading expansion): All: (antibacterial drugs) *All: (use)* All: (behavior)* All:(Population) (In Chinese)
VIP Information/Chinese Scientific Journals database	(U=(antibacterial drug OR antibiotic OR antimicrobial drug)) AND (U=(use OR application OR management OR storage OR Self-purchase OR Drug Purchase Without Prescription OR Drug Purchase) AND (U= (Current Situation OR Behavioral Factors OR Knowledge OR Attitude OR Reasonable OR Unreasonable OR Abuse OR Expectation OR Doctor’s Order OR Compliance)) AND (U= (Resident OR The general population OR the public OR humans OR respiratory tract infection patients OR students OR middle-aged and elderly patients OR outpatients)) (In Chinese)
Chinese Biomedical Literature Service System	#1. "Antibacterial Drugs" [Common Fields: Smart] OR "Antimicrobial Drugs " [Common Field: Smart] OR "Antibiotic"[Common Field: Smart];<br><br>#2. "Use" [Common Field: Smart] OR "Application" [Common Field: Smart] OR "Management" [Common Field: Smart] OR "Storage"[Common Field: Smart] OR "Purchase Medicine by Yourself"[Common Field: Smart] OR "Purchase Medicine Without Prescription"[Common Field: Smart];<br><br>#3. "Current Situation"[Common Field: Smart] OR "Behavioral Factors" [Common Field: Smart] OR "Cognition" [Common Field: Smart] OR "Attitude" [Common Field: Smart] OR "Reasonable" [Common Field: Smart] OR "Expectation" [Common Field: Smart]; OR "Doctor's Advice"[Common Field: Smart] OR "Compliance"[Common Field: Smart] OR "Behavior"[Common Field: Smart];<br><br>#4. "population" [common field: smart] OR "resident" [common field: smart] OR "general population" [common field: smart] OR "public"

[common field: smart] OR "human" [common field :Smart] OR "Patients with Respiratory Tract Infection"[Common Field: Smart] OR "Middle and Elderly"[Common Field: Smart] OR "Student"[Common Field: Smart] OR "Outpatient"[Common Field: Smart];

#5. (#1) AND (#2) AND (#3) AND (#4) (In Chinese)

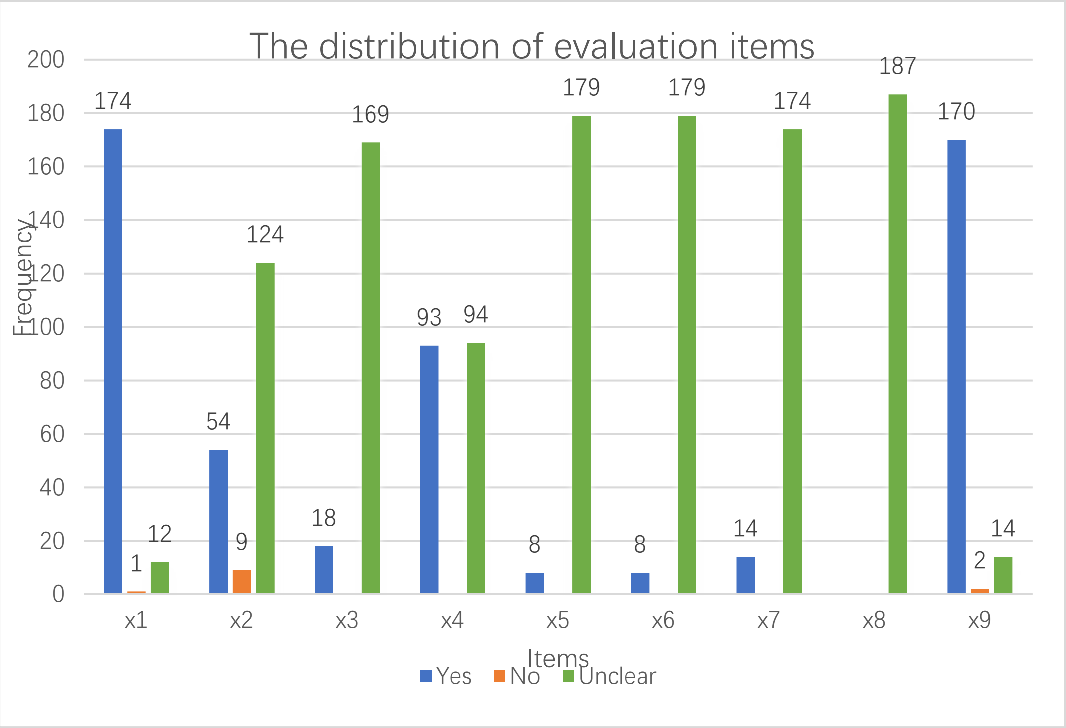

Figure S1. The distribution of evaluation items

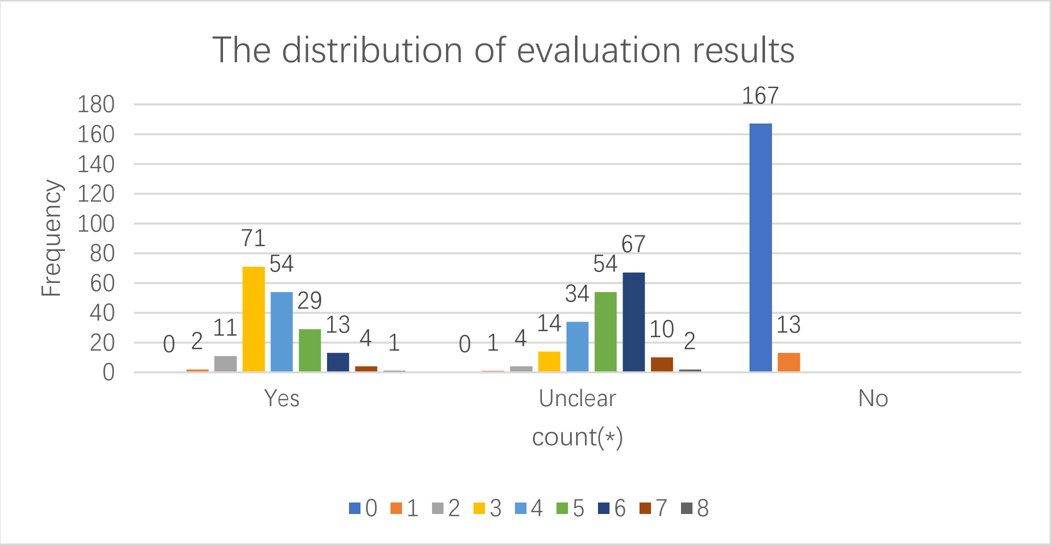

Figure S2. The distribution of assessment score

Appendix A JBI Critical Appraisal Checklist for Studies Reporting Prevalence Data

Reviewer____________________ Date_____________________________

Author_______________ Year ________ Record Number___________________

	Yes	No	Unclear	Not applicable
1.Was the sample frame appropriate to address the target population?	?	?	?	?
2.Were study participants sampled in an appropriate way?	?	?	?	?
3.Was the sample size adequate?	?	?	?	?
4.Were the study subjects and the setting described in detail?	?	?	?	?
5.Was the data analysis conducted with sufficient coverage of the identified sample?	?	?	?	?
6.Were valid methods used for the identification of the condition?	?	?	?	?
7.Was the condition measured in a standard, reliable way for all participants?	?	?	?	?
8.Was there appropriate statistical analysis?	?	?	?	?
9.Was the response rate adequate, and if not, was the low response rate managed appropriately?	?	?	?	?

Overall appraisal: Include: ? Exclude : ? Seek further info: ?

Comments (Including reason for exclusion)

© Joanna Briggs Institute 2016

Explanation of Prevalence Critical Appraisal

1. Was the sample frame appropriate to address the target population? The sampling frame should match with the purpose of the study, and can obtain the target population of the study;

2. Were study participants sampled in an appropriate way? It is necessary to describe in detail what sampling method was used and how to gradually obtain our target population. For cluster and stratified sampling, you should describe the characteristics according to which participants are stratified and grouped. Non-probability sampling is not considered an appropriate survey research method;

3. Was the sample size adequate? In addition to a comprehensive census, a detailed and reasonable sample size calculation process is required for sample surveys;

4. Were the study subjects and the setting described in detail? The characteristics of the participants need to be described in detail, and demographic information, research time and research location need to be included at the same time;

5. Was the data analysis conducted with sufficient coverage of the identified sample? It is necessary to describe whether the samples can adequately cover the structure of each subgroup;

6. Were valid methods used for the identification of the condition? While using a self-made questionnaire as measurement tool, the reliability and validity test results of the questionnaire need to be provided; if a certain established standard or definition is applied in the research, its standard and source should be introduced in detail;

7. Was the condition measured in a standard, reliable way for all participants? introduce whether the questionnaire surveyor has been uniformly trained, and introduce the content of the uniform training, or describe in detail the distribution and quality control process of the questionnaire in the data collection process.

8. Was there appropriate statistical analysis? The rate index should give the numerator, denominator, and rate with confidence interval.

9. Was the response rate adequate, and if not, was the low response rate managed appropriately? The response rate of the research needs to be given. Generally, the response rate is >70%. If the response rate is less than 70%, the response rate is too low. The research needs to provide solutions for the response rate.

Appendix B Data extraction form for prevalence studies

Citation Details						
Author	Title	Journal	Year	Issue	Volume	Pages

Generic Study details						
Study design	Country	Setting/Context	Year/timeframe for data collection	Participants Characteristic (study inclusion/exclusion information)	Condition and measurement	Description of main result(n/N)
